# User Experience in Remote Surgical Consultation: Survey Study of User Acceptance and Satisfaction in Real-Time Use of a Telemedicine Service

**DOI:** 10.2196/30867

**Published:** 2021-11-30

**Authors:** Hedvig Aminoff, Sebastiaan Meijer, Kristina Groth, Urban Arnelo

**Affiliations:** 1 Department of Biomedical Engineering and Health Systems School of Engineering Sciences in Chemistry, Biotechnology and Health KTH Royal Institute of Technology Stockholm Sweden; 2 The Center for Innovation Karolinska University Hospital Stockholm Sweden; 3 CLINTEC Department of Clinical Science, Intervention and Technology Karolinska Institutet Stockholm Sweden; 4 Department of Surgical and Perioperative Sciences Umeå University Umeå Sweden

**Keywords:** telemedicine, user experience, satisfaction, technology acceptance, usability, perioperative, surgery, consultation, surgeons, performance, evaluation, teleguidance, telehealth, telemedicine implementation

## Abstract

**Background:**

Teleguidance, a promising telemedicine service for intraoperative surgical consultation, was planned to scale up at a major academic hospital in partnership with 5 other hospitals. If the service was adopted and used over time, it was expected to provide educational benefits and improve clinical outcomes during endoscopic retrograde cholangiopancreatography (ERCP), which is a technically advanced procedure for biliary and pancreatic disease. However, it is known that seemingly successful innovations can play out differently in new settings, which might cause variability in clinical outcomes. In addition, few telemedicine services survive long enough to deliver system-level outcomes, the causes of which are not well understood.

**Objective:**

We were interested in factors related to usability and user experience of the telemedicine service, which might affect adoption. Therefore, we investigated perceptions and responses to the use and anticipated use of a system. Technology acceptance, a construct referring to how users perceive a technology’s usefulness, is commonly considered to indicate whether a new technology will actually be used in a real-life setting. Satisfaction measures were used to investigate whether user expectations and needs have been met through the use of technology. In this study, we asked surgeons to rate the perceived usefulness of teleguidance, and their satisfaction with the telemedicine service in direct conjunction with real-time use during clinical procedures.

**Methods:**

We designed domain-specific measures for perceived usefulness and satisfaction, based on performance and outcome measures for the clinical procedure. Surgeons were asked to rate their user experience with the telemedicine service in direct conjunction with real-time use during clinical procedures.

**Results:**

In total, 142 remote intraoperative consultations were conducted during ERCP procedures at 5 hospitals. The demand for teleguidance was more pronounced in cases with higher complexity. Operating surgeons rated teleguidance to have contributed to performance and outcomes to a moderate or large extent in 111 of 140 (79.3%) cases. Specific examples were that teleguidance was rated as having contributed to intervention success and avoiding a repeated ERCP in 23 cases, avoiding 3 PTC, and 11 referrals, and in 11 cases, combinations of these outcomes. Preprocedure beliefs about the usefulness of teleguidance were generally lower than postprocedure satisfaction ratings. The usefulness of teleguidance was mainly experienced through practical advice from the consulting specialist (119/140, 85%) and support with assessment and decision-making (122/140, 87%).

**Conclusions:**

Users’ satisfaction with teleguidance surpassed their initial expectations, mainly through contribution to nontechnical aspects of performance, and through help with general assessment. Teleguidance shows the potential to improve performance and outcomes during ERCP. However, it takes hands-on experience for practitioners to understand how the new telemedicine service contributes to performance and outcomes.

## Introduction

### Overview

A telemedicine service for intraoperative surgical consultation during advanced gastrointestinal endoscopy was seen as having the potential to increase the quality and safety of procedures, and there was hope that the technique could be used in other areas of medicine. The service, called teleguidance, had shown success in a feasibility study [1], and health economic modeling showed its potential for improving clinical and economic outcomes [2]. Teleguidance practice was to be scaled up to 4 additional hospitals (5 in total including the first hospital), and efforts were made to understand the context into which the telemedicine intervention was introduced [3], and potential users’ attitudes toward the service prior to implementation [4]. This paper describes an investigation of practitioners’ experience of real-time use of teleguidance, based on surgeons’ expectations of how the service might contribute to performance and outcomes in a particular procedure, and their satisfaction with teleguidance immediately after the procedure.

### Background

Teleguidance is a professional-to-professional telemedicine service for video collaboration during a highly specialized endoscopic procedure called endoscopic retrograde cholangiopancreatography (ERCP).

ERCP is a technically advanced procedure for examination, sampling, and interventions in the complicated ductal structures of the gall bladder, pancreas, and liver; for example, to remove or alleviate blockages caused by tumors in the liver. Today, ERCP is a common procedure, and when successful, it can quickly relieve very painful and serious conditions; it is sometimes a prerequisite for consecutive procedures. However, ERCP is a complex, high-risk, collaborative task and a highly technical specialty with a long learning curve. Learning the perceptual and motor skills to control the equipment and interpreting the guiding video and x-ray images requires considerable practice and skill, combined with clinical decision-making based on careful weighing of risks and benefits.

Clinical practitioners had expressed positive expectations that teleguidance could contribute to the quality and safety of procedures but also concerns that teleguidance might disrupt work practices [4]. The telemedicine service was designed through participatory, user-centered development [5], and there were hopes that by providing remote intraoperative consultation, teleguidance could contribute to learning and improved performance, which could enable practitioners at smaller hospitals to provide more highly specialized procedures. A feasibility study had reported clinical benefits [1], and health economic modeling showed the potential for positive clinical and economic outcomes [2]. A decision was made to scale up teleguidance to 4 additional hospitals, with an intention to generate additional evidence for the benefits of the practice.

### The Need for Expertise in ERCP

Increasing therapeutic use of ERCP and increasing procedural complexity has raised the level of expertise required for ERCP [6]. At smaller hospitals in Sweden, many individual ERCP practitioners and clinics have an annual procedural volume which is below the recommendations for sustaining and advancing skill [7].

Traditionally, advanced surgical skills are learned by working together with experienced surgeons, progressing from shadowing to increasingly independent work with hands-on training and mentorship. Once proficiency is gained, a certain procedural volume is generally considered necessary to sustain newly acquired skills, develop experience, and keep up with new technical advances, and high procedural volume is associated with fewer adverse events [8].

Studies have shown that there are large variations in the quality of ERCP procedures at different clinics [9], and failure to cannulate the desired duct or post-ERCP pancreatitis is common but has serious consequences [10]. Repeated unsuccessful attempts to cannulate the correct duct play a significant role for complications [11], and cannulation failure can lead to a decision to abort a procedure, causing a subsequent delay in treatment, or conversion to more invasive procedure, such as percutaneous transhepatic cholangiography (PTC) [12]. Post-ERCP pancreatitis and other serious adverse events such as bleeding or perforation, have been found to be more common when the procedure was performed by less experienced practitioners [13].

It has been suggested that ERCP specialists with lower levels of expertise should not attempt complex or difficult ERCP cases without the assistance of a more experienced endoscopist [12], and that serious outcomes can be avoided if there is an option to cooperate with other highly specialized colleagues in the case of adverse events [6].

At larger hospitals, a practitioner needing advice during a difficult procedure often has a colleague ERCPist on call to advise or assist during a difficult procedure. This is not always the case at smaller hospitals, and practitioners have commonly used the telephone when they needed advice. Remote surgical guidance in ERCP, *teleguidance*, via videoconferencing and simultaneous transfer of high-quality surgical imaging was developed to enhance this practice to help develop and sustain the expertise of individual practitioners at lower-volume centers [1].

### Remote Surgical Consultation and Mentoring

Services similar to teleguidance, such as surgical telementoring and remote surgical guidance, have previously been developed to support education and address knowledge gaps [14-16] and increase access to highly specialized treatment in remote or low-case-load facilities [17-20]. They have, for example, been used in trauma and emergency medicine [21] and in laparoscopic and open surgery [22].

However, for the potential benefits of any telemedicine service to be fulfilled over time, a service also needs to become a part of regular practice [23]. Despite a wide range of apparent benefits, there is limited evidence for the educational benefits of telementoring [15], and this way of working has generally failed to become a daily tool in clinical workflows [15,16,18,24]. While a wide range of barriers to telemedicine implementation and adoption have been identified [25], how these individual factors contribute to assimilation and sustainable use of telemedicine in real-life practice is not well understood [26]. However, feedback about real-time use of telemedicine systems can be a way to gain a better image of the factors that can affect clinical outcomes and adoption [27-29].

In evaluations of the impact of telemedicine services on clinical outcomes, telemedicine can be defined as a complex intervention [30]; as such, there is value in complementing traditional clinical assessments of effectiveness with qualitative studies of user perceptions and experience [29,31]. According to ISO 9241-11, usability is the “extent to which a system, product or service can be used by specified users to achieve specified goals with effectiveness, efficiency and satisfaction in a specified context of use” [32]. Usability evaluation can include combinations of objective measures of effectiveness (eg, successful task completion rate) and efficiency (eg, task completion time), and subjective measures of satisfaction [33], which can be a valuable a part of an effort to understand the likelihood of acceptance and use of a telemedicine system.

### Perceived Usefulness and Satisfaction

Telemedicine research has shown that users’ perceptions of a technology’s usefulness are a main significant predictor of acceptance [26]. The concept of technology acceptance is widely used in telemedicine research [26] but originated in information systems research [34], where behavioral theory and methodologies have generated a large body of research about how users’ attitudes toward a technology influence subsequent adoption and use [35]. The technology acceptance model (TAM) [36] conceptualizes acceptance as an evaluative process, where technology use can be predicted or explained on the basis of psychometric measures of users’ expectations about perceived usefulness; that is, how using a technology will impact job effectiveness, efficiency, and performance [37].

Satisfaction is also a considered a key component for telemedicine success and is often included in the evaluation of telemedicine services [38]. Telemedicine satisfaction studies have often reported favorable results [39,40]; nonetheless, methodological issues often make it difficult to interpret or compare findings [41], and it is often unclear what satisfaction measures actually demonstrate [42]. In health services research, satisfaction measures may implicitly refer to patient satisfaction with treatment or care [43,44], which contrasts with measures of satisfaction with the use of a technology; that is, whether user expectations and needs have been met through the use of technology [32,35,45,46].

This indicates a need for careful definition and operationalization of measures of perceived usefulness and satisfaction when collecting feedback about the real-time use of telemedicine, to ensure a match between the constructs and the technology involved, the users, and the context in which the telemedicine service is being introduced.

### Aims and Objectives

Telemedicine acceptance and adoption are not well understood [47,48], and many telemedicine services fail to be adopted, despite their apparent value; this lack of successful implementation is so common it has been described a “paradox of telehealth” [49-51]. However, information systems research has shown that users’ expectations of a technology’s usefulness is an important determinant for adoption, and that satisfaction measures can be used to investigate whether user expectations of using a technology have been met.

When teleguidance was scaled up, we were interested in how clinical practitioners experienced the usefulness of the telemedicine service, and if the service lived up to these expectations, as this could provide insight into the factors shaping the success of the intervention. We were provided with an opportunity to gather data in direct conjunction with teleguidance sessions, and wanted to investigate whether clinical practitioners expected teleguidance to contribute to a specific case, and whether these expectations were met during the teleguidance session. These subjective measures of anticipated usefulness and satisfaction with using teleguidance are expected to provide knowledge about central user perceptions and user experience, which can influence the implementation, adoption, and use of remote surgical consultation.

## Methods

### Methods Overview

Participating surgeons at the central hospital and the 5 participating remote sites filled in case report forms (CRF) in conjunction with each teleguided ERCP procedure. These data were passed in raw form to us. In the following sections, the design of the CRFs and the rationale for data collection, which underlies how we operationalized the constructs perceived usefulness and satisfaction, is described.

### Design of the CRF

Two paper-based CRFs were designed to gather pre- and postprocedure data. The guiding surgeon at the central site registered patient- and case-related data, mainly to ensure correct orientation about the case to be guided and to provide ratings of the technical quality of image transmission. The remote surgeons were asked to register patient- and intervention-related data and a rating of the level of complexity of each case. In addition, they were asked to provide subjective ratings of their estimated need and expectations for consultation during the procedure, and to report technical issues and their experience of how teleguidance contributed to performance and outcomes.

#### The Guiding Surgeons’ CRF

Prior to a teleguidance session, the guiding specialist requires basic information about the case and the patient, and this was communicated either by telephone or through the videoconferencing system. The guider was asked to register the data necessary to ensure a correct understanding of the needs and potential risks during consultation: if it is an emergency or planned elective procedure, the patient’s gender, age, and whether ERCP has been performed previously. In addition, knowledge about whether the remote procedure was to be conducted with sedation or general anesthesia provided information about the patient’s orientation, which has consequences regarding the interpretation of the transmitted image. The indications and aims of the procedure provided fundamental information to the guiding specialist about what was to be done during the ERCP.

#### The Remote Surgeons’ CRF

The CRF contained data entry fields about clinical indications, the aims and success of the procedure, and 30-day follow-up items about complications, consecutive procedures, and health economic data. The CRF also included 2 subjective preprocedure rating items about the benefits the participants were hoping for and the problems they were hoping to avoid through teleguidance. After teleguided procedures, satisfaction with teleguidance was measured through participants’ ratings of the ways in which teleguidance contributed to their performance and to the outcomes of the procedure.

Appropriate indication, cannulation rate, stone extraction success rate, stent insertion success rate, and post-ERCP pancreatitis frequency are evidence-based, prioritized quality indicators for ERCP [12]. Therefore, we considered these factors relevant for perceived usefulness and investigated whether participants believed that teleguidance could contribute to cannulation, stone extraction, and stent insertion. We also investigated whether practitioners considered teleguidance to support clinical assessment and decision-making, and help avoid additional interventions or referrals.

Performance and outcome measures such as cannulation frequency or complication frequency are not straightforward to interpret: more complex cases generally have a higher risk for complications [13]. Therefore, we also wanted to obtain information about the clinical difficulty of the procedure. This was measured through a preprocedure case complexity rating, with 4 predefined categories of clinical contexts, techniques, and anatomical or pathological features (Multimedia Appendix 1).

Participants were also asked to grade cannulation difficulty after each teleguided procedure in accordance with the 5-5-2 principle defined by the European Society of Gastrointestinal Endoscopy [52]. The number of earlier ERCPs the patient has undergone and the characteristics of the papilla are also associated with cannulation outcomes [53] and were therefore also included in the CRF.

Multimedia Appendix 2 shows the CRF items related to perceived usefulness, prior to teleguidance sessions, and satisfaction immediately experience after teleguided sessions.

### Procedure

Each participating hospital received a utility cart equipped with the necessary components to transmit endoscopic and fluoroscopic images. The cart was also equipped with a camera and microphone to capture images and sounds from the operation theater.

The teleguidance equipment had one video and one content channel, which meant that the participants had to choose from among endoscopy, fluoroscopy, or a view of the operating room. The remote party controlled switching between imaging, and would change upon request from the consulting surgeon. Audio communication was possible throughout the session.

**Figure 1:**
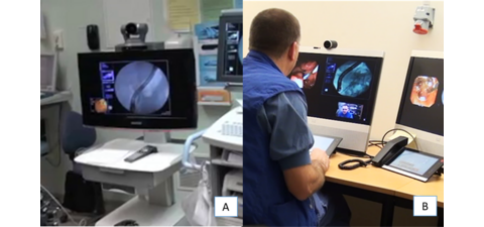
The remote clinics used a Polycom Realpresence Practitioner cart 8000 (A). At the central site, the consulting surgeon used a Polycom HDX4500 desktop video conferencing system with a touch screen control (B).

The remote sites used the following teleconference systems: a mobile Polycom Practitioner Cart HDX unit (Polycom) equipped with a 26-inch LCD screen, a high-definition video camera, stereo speakers, and microphones (Figure 1). At the University hospital, a Polycom HDX 4500 desktop videoconference system was used to provide guidance from either an office near to the endoscopy suite in which the ERCP interventions were usually performed, or an office equipped with multiple videoconference systems set up specifically for teleguidance.

All communication passed through Sjunet, a secure, IP-based broadband network for Swedish health care providers (Sjunet).

There was no function for telestration (annotating live video content telestration) during scaling up of teleguidance. During the design of the teleguidance solution, a prototype for telestration (Multimedia Appendix 3) had been tested in a few scenarios. It was hoped that a function for graphical annotation would improve shared understanding in tasks such as localization of the point of entry to the common bile duct. However, the results indicated that users on both ends of the teleguidance session were distracted by the design and function of this particular telestration solution. Our findings echoed those of a more well-designed study [54], and a decision was made not to further develop this function.

Written instructions, contact details to the guiding practitioners, and technical support, and a protocol for establishing a connection among the hospitals were also provided (Multimedia Appendix 4). Clinical and technical staff received a tutorial.

The operating surgeons and the consulting surgeon used the telephone to agree on the timing for the teleguidance session days ahead, or in some cases, immediately before a procedure. The remote sites initiated the teleguidance sessions.

CRFs were distributed to each site to be filled in on paper by the operating endoscopist and submitted to a coordinating research nurse at the central site.

Participants were instructed to teleguide as many ERCP cases as possible during the study period and not to select cases. Operating surgeons were asked to book teleguidance sessions in advance by telephone, but there was also the option to call in direct conjunction with a procedure.

Two senior ERCP experts at a high-volume tertiary referral ERCP clinic provided remote consultation via teleguidance.

### Sample

ERCP procedures at 5 district hospitals received teleguidance from a tertiary referral center. In total, 142 teleguided procedures are included in the sample. The average duration of ERCP procedures was 53 minutes (range 10-224 minutes, median 45 minutes). The average duration of teleguidance sessions was 43 minutes (range 4-186 minutes, median 35 minutes).

The most common indications were biliary and pancreatic stones (56/142, 39%) and icterus (33/142, 23%); the most common aim for procedures were ERC stone extraction (71/142, 50%) and ERC offload (59/142, 42 %) (Multimedia Appendix 5). A total of 75 of 142 (53%) patients were female, and 67 of 142 (47%) were male. The age range was between 18 and 91 years, the mean age of female patients was 67 years and that of men was 67 years. Furthermore, 43 of 142 (30%) cases were emergency interventions, while 93 of 142 (66%) were elective (5/142, 4% were not classified). Additional details about the patients and case complexity ratings are shown in Multimedia Appendix 6.

In total, 14 ERCPists participated at the remote sites. Table 1 shows the level of experience among participants at the remote sites. All 5 novices with low experience (>200 ERCP) progressed to an expert level of 500-1000 ERCP procedures during this period. The distribution of cases across hospitals and practitioners is shown in Multimedia Appendix 7.

**Table 1:** Level of experience among participants at remote sites (n=11).

Participants	Guided sessions (n=142), n (%)
Novice (<200 ERCP^a^)	14 (9.9)
Novice (200-500 ERCP)	33 (23.2)
Expert (500-1000 ERCP)	87 (61.3)
Expert (>1000 ERCP)	8 (5.6)

^a^ERCP: endoscopic retrograde cholangiopancreatography.

The remote sites reported technical issues in 26 of 142 (11%) cases; however, these were problems experienced by the consulting surgeon at the central site. This did, however, cause inconvenience at the remote sites, since the remote sites had to conduct troubleshooting in these cases.

The central site reported technical issues in 34 of 142 (24%) cases. In total, 16 of 34 (47%) of the reported problems regarded acoustic feedback between microphones on the teleguidance cart and microphones in the operation theater at one of the hospitals. In 9 of 34 (26%) cases, there were problems with pixelated image quality or problems with hue. In 5 of 34 (15%) cases, the consulting surgeon could see the endoscopic video, but there were intermittent problems with transfer on fluoroscopy.

In some cases, this was resolved by restarting the connection; in some cases, medical technicians at the remote sites provided assistance and resolved problems; for example, by changing video graphics array cables between monitors. While this caused some delays, the teleguidance sessions proceeded despite the technical issues. There were 4 cases of postoperative complications (4/142, 2.8%).

## Results

### Perceived Usefulness of Teleguidance

**Figure 2:**
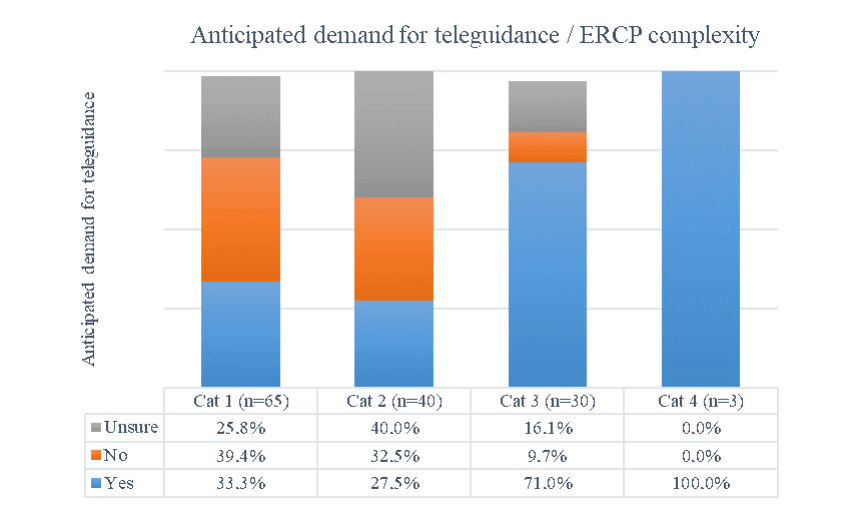
Anticipated demand for teleguidance/endoscopic retrograde cholangiopancreatography complexity. ERCP: endoscopic retrograde cholangiopancreatography.

Perceived usefulness was measured on the basis of surgeons’ ratings of their expectations of how teleguidance might contribute to procedures. Table 2 and Figure 2 show how surgeons rated their anticipated demand for teleguidance prior to specific procedures: in 58 of 139 (41%) procedures, surgeons expected to have use for teleguidance; 42 of 139 (30%) reported that they did not; and 38 of 139 (27%) were unsure.

In 20 cases, the option to use teleguidance had affected the decision to perform the procedure in question.

The operating surgeon’s level of expertise did not significantly affect the anticipated demand for teleguidance (*r*=0.008, *P*>.001) (Multimedia Appendix 8).

**Table 2:** Anticipated demand for teleguidance.

Response	Surgeons, n (%)
Yes	58 (42.0)
No	42 (30.4)
Unsure	38 (27.5)

However, the demand for teleguidance was more pronounced in cases with higher complexity according to the 4-category rating scale (Multimedia Appendix 1). Case complexity and the anticipated demand for teleguidance (Table 2) showed a significant linear relationship (*r*=–0.229, *P*<.001).

Participants expressed a higher anticipated demand for teleguidance for certain intervention goals; however, we did not observe any significant relationships. In ERC stone extractions, only 21/69 (30%) rated a need for teleguidance while there was more demand in the other procedures, especially regarding pancreatic procedures: ERC offload (28/58, 47.5%), endoscopic retrograde cholangiography (ERC) biopsy (12/21, 57%), and the pancreatic procedures ERP stone extraction (6/11, 54.5%) and ERP offload (8/11, 72.7%) (Multimedia Appendix 9).

Similarly, participants expressed a higher anticipated demand for teleguidance for certain indications (Multimedia Appendix 10). The demand for teleguidance was high (>40%) for acute pancreatitis (7/15, 46.7 %), chronic pancreatitis (10/12, 83.3%), primary sclerosing cholangitis (PSC) (5/5, 100%), and strictures with unknown causes (5/9, 55.6%).

In 82 of 142 (57.7%) cases, surgeons reported expectations to avoid certain situations through teleguidance (Multimedia Appendix 11). The most frequent situation they hoped to avoid was having to repeat the ERCP, which is commonly owing to a failure to cannulate (42/82, 51.2%).

In 67 of 142 (53%) cases, the surgeons expressed an expectation to receive support with specific tasks during the procedure (Multimedia Appendix 12). In total, 10 of 67 (14.9%) hoped to receive support with cannulation; 4 of 67 (6%) hoped to receive support with the placement of a stent; 6 of 67 (9%) hoped to receive support with the removal of a stone; 27 of 67 (34%) hoped to receive support with clinical assessment; and 21 of 72 (28%) hoped to receive support with combinations of these tasks.

### Satisfaction With Teleguidance

Satisfaction was measured after procedures, through ratings of how teleguidance contributed to performance and outcomes.

The operating endoscopists rated teleguidance to have contributed value to a moderate or large extent (rating value 3 and 4) in 111 of 140 (79.3%) cases (Multimedia Appendix 13). In 16 of 26 (61%) cases where the operating surgeon reported technical problems, this rating was somewhat lower.

**Figure 3:**
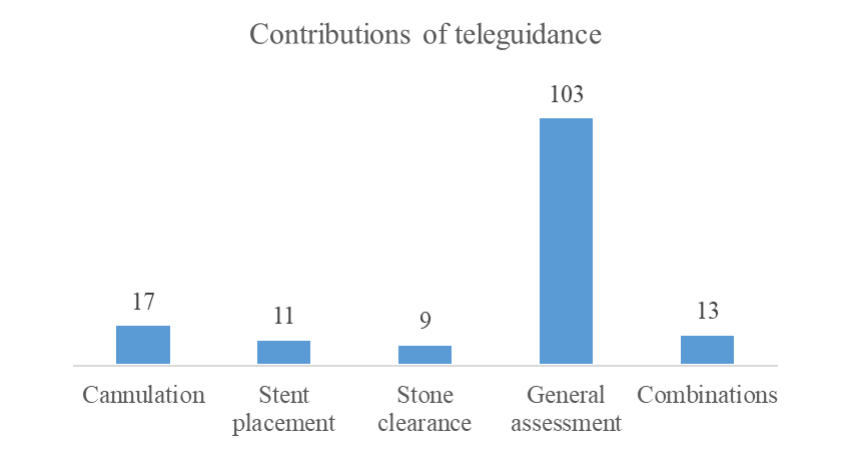
Contributions of teleguidance.

Teleguidance was rated as having contributed to cannulation in 17 of 140 (11.9%) cases, and classified as difficult cannulations in 11 of 39 (28%) cases. In 11 of 140 (7.7%) cases, teleguidance was rated as having contributed to stent placement, to stone clearance in 9 of 140 (6.3%) cases, to general assessment in 103 of 140 (72%) cases, and to combinations of these contributions in 13 of 140 (9.3%) cases (Figure 3).

In 23 of 140 cases, surgeons considered teleguidance to have contributed to intervention success and avoiding a repeated attempt at the same intervention (re-ERCP). In 3 of 140 cases, PTC—a more painful and invasive procedure than ERCP—was avoided. In 11 of 140 cases, referral to another ERCP center could be avoided, and in 11 of 140 cases, combinations of the above, and 3 of 140 unspecified other interventions could be avoided (Table 3).

**Table 3:** Procedures avoided owing to teleguidance (N=140).

Procedures avoided	Cases, n (%)
Re-ERCP^a^	23 (16.1)
Percutaneous transhepatic cholangiography	3 (2.1)
Other intervention	3 (2.1)
Referral	11 (7.7)
Combinations	11 (7.7)

^a^ERCP: endoscopic retrograde cholangiopancreatography.

**Figure 4:**
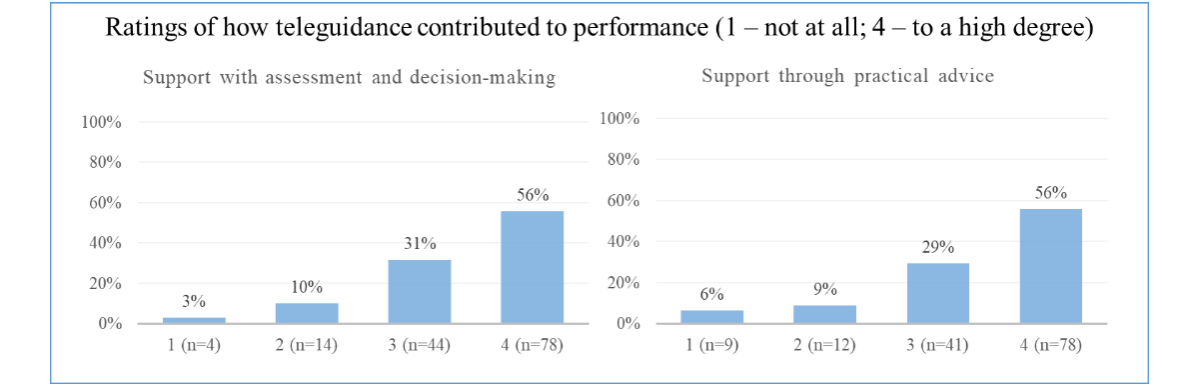
Teleguidance provided support through practical advice and help with assessment.

In 119 of 140 (85%) cases, teleguidance was reported as having contributed through practical advice to a moderate or large extent, and 122 of 140 (87%) reported that they received support with assessment and decision-making during the procedure to a moderate or large extent (Figure 4).

Overall, the satisfaction ratings, which were measured after procedures, were higher than perceived usefulness ratings, which were measured prior to procedures.

## Discussion

When a telemedicine service for intraoperative surgical consultation was scaled up, we were interested in users’ perceptions of how the service contributed to performance and outcomes, as this might provide insight into adoption and use of the telemedicine service over time. We designed and collected measures of perceived usefulness and satisfaction in direct conjunction with real-time use in ERCP procedures. The measures were intended to reflect how users considered teleguidance to contribute to performance and outcomes during ERCP procedures.

Practitioners believed that teleguidance would be useful; that is, as having value for performance and outcomes, prior to a high proportion of cases. In roughly half of the cases, surgeons specified the type of support they expected, which, in most cases, was related to clinical assessment (27/67, 40.3%). However, the anticipated demand for teleguidance increased with the level of procedural complexity (Multimedia Appendix 14), and there was more interest for teleguidance in certain clinical indications, such as acute and chronic pancreatitis, PSC, and strictures of unknown type (Multimedia Appendix 11).

The results showed that less experienced practitioners perceived teleguidance as more useful than their experienced colleagues did, but the findings were not significant. In addition, the perceived usefulness of teleguidance was higher in cases that could be expected to be challenging.

Regarding satisfaction with teleguidance after procedures, the operating endoscopist rated teleguidance to have contributed to performance and outcomes to a moderate or large extent in 111 of 140 (79.3%) cases. Specific examples are as follows: contribution to intervention success and avoiding a repeated ERCP in 23 cases, avoiding 3 PTC, and 11 referrals, and in 11 cases, combinations of these outcomes (Multimedia Appendix 15).

**Figure 5:**
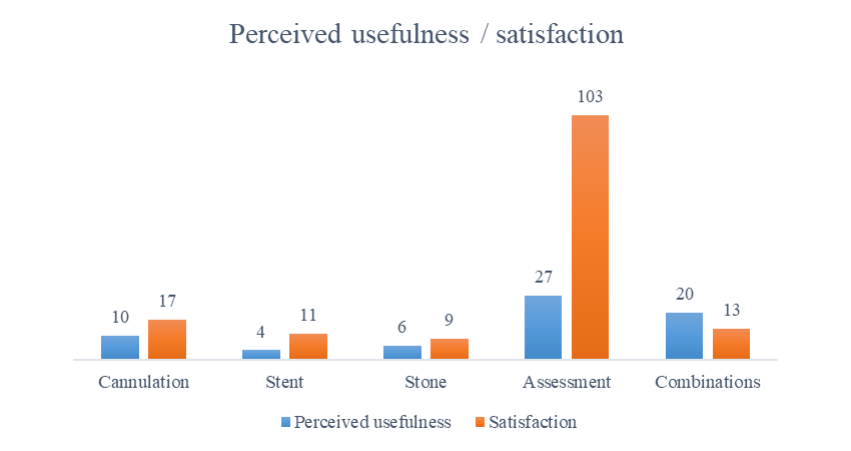
Our results show that satisfaction after using teleguidance was higher than beliefs about usefulness prior to procedures.

Our results show that satisfaction after using teleguidance was higher than the preprocedure usefulness of teleguidance, which was rated prior to the procedures (Figure 5). This indicates that it is difficult for practitioners to predict how a novel way of working, such as teleguidance, can contribute to performance and outcomes. User beliefs and attitudes toward technology can be expected to change with first-hand use [55]. Our findings indicate that doctors may become more cognizant of how remote surgical consultation can support important clinical and development/training aspects in ERCP [3] with hands-on use, but also that they require some time before they assimilate teleguidance into their practice. The technical issues experienced did cause some inconvenience and delays, but did not appear to cause teleguidance sessions to be terminated. However, the satisfaction ratings were lower in the cases where technical issues were encountered.

While training and assessment of surgical performance is commonly focused on technical ability, cognitive and social skills are also important requirements for surgical competence. Teleguidance may be of value for these nontechnical skills for surgeons, which have been defined as “behavioral aspects of performance in the operating room which underpin medical expertise, use of equipment and drugs: cognitive (e.g. situation awareness, decision making) social (e.g. communication & teamwork, leadership) skills” [56].

Research has shown that user beliefs and attitudes toward technology can be expected to change with first-hand use [55]: experience of teleguidance may gradually change over time. This study also only focused on clinicians’ experience of teleguidance during procedures and does not consider contextual factors that are believed to affect the acceptance of teleguidance [3]. Therefore, these results represent an interim judgement of usefulness and satisfaction, which may differ from final overall satisfaction outcomes [35].

### Conclusions

Surgeons appeared not to have expected the level of support they received through remote surgical consultation during ERCP. They also received help with surgical/technical tasks, such as stent placement and stone removal. Each case of support may be of high value from a patient’s perspective and for ERCP quality and health economic reasons. The difference between preprocedure expectations and postprocedure satisfaction indicates that practitioners require hands-on use experience to understand the usefulness of the new telemedicine service and how it contributes to surgical procedures. For this reason, adoption can be expected to develop over time and require extended use before being accepted.

While a larger sample of procedures is required to be able to draw statistical inferences about the contribution of teleguidance on clinical outcomes, the findings from the survey items on perceived usefulness and satisfaction indicate that surgeons consider teleguidance to contribute to nontechnical aspects of surgical performance, such as decision-making, to an extent that many practitioners did not anticipate.

This study represents part of a human-centered approach to system design, where system quality is linked to how well users can achieve specific goals. From a methodological perspective, it would be interesting to investigate how interim measures of acceptance and satisfaction correspond with final acceptance and use of the telemedicine service, which appears to be a lesser investigated area [35]. In the case of teleguidance, the service was largely abandoned after the initial start-up phase, even though it is intermittently used in-house at the central hospital.

This study also indicates a need to more deeply investigate how remote surgical contributes to clinical procedures, and the ways in which this way of working differs from on-site surgical consultation.

## Appendix

Multimedia Appendix 1 Endoscopic retrograde cholangiography (ERCP) complexity rating.

Multimedia Appendix 2 Case report form (CRF) items relating to perceived usefulness and satisfaction.

Multimedia Appendix 3 A protoype for telestration was tested during development but not included in the solution that was used in this study.

Multimedia Appendix 4 Protocol for establishing a connections between the hospitals.

Multimedia Appendix 5 Indications and aims for the procedures.

Multimedia Appendix 6 Patient sex and age, and case complexity ratings.

Multimedia Appendix 7 Distribution of cases across hospitals and practitioners.

Multimedia Appendix 8 The operating surgeon’s level of expertise and the demand for teleguidance.

Multimedia Appendix 9 Intervention goals and anticipated demand for teleguidance.

Multimedia Appendix 10 Anticipated demand for teleguidance for different indications.

Multimedia Appendix 11 Situations which surgeons believed teleguidance might help them might avoid.

Multimedia Appendix 12 Tasks that surgeons expected to get support with through teleguidance.

Multimedia Appendix 13 Ratings of the value of teleguidance.

Multimedia Appendix 14 A higher level of case complexity corresponded with a higher demand for teleguidance.

Multimedia Appendix 15 In many cases, practitioners considered teleguidance to have have helped avoid unwanted outcomes.

## Abbreviations

CRF: case report form
ERC: endoscopic retrograde cholangiography
ERCP: endoscopic retrograde cholangiopancreatography
PSC: primary sclerosing cholangitis
PTC: percutaneous transhepatic cholangiography
TAM: technology acceptance model
