# Supplementary figures and images for: User Experience in Remote Surgical Consultation: Survey Study of User Acceptance and Satisfaction in Real-Time Use of a Telemedicine Service
Source: JMIR Hum Factors. 2021 Nov 30;8(4):e30867. doi: 10.2196/30867 (PMC8672288; doi:10.2196/30867)

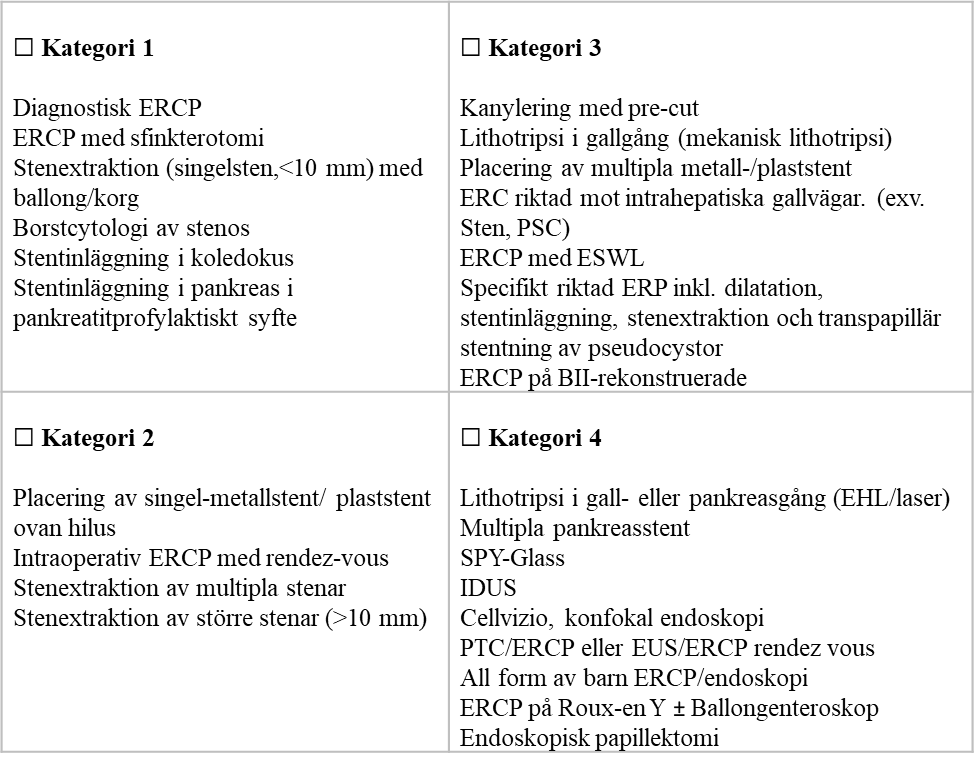

Supplement: Multimedia Appendix 1 [file humanfactors_v8i4e30867_app1.png]

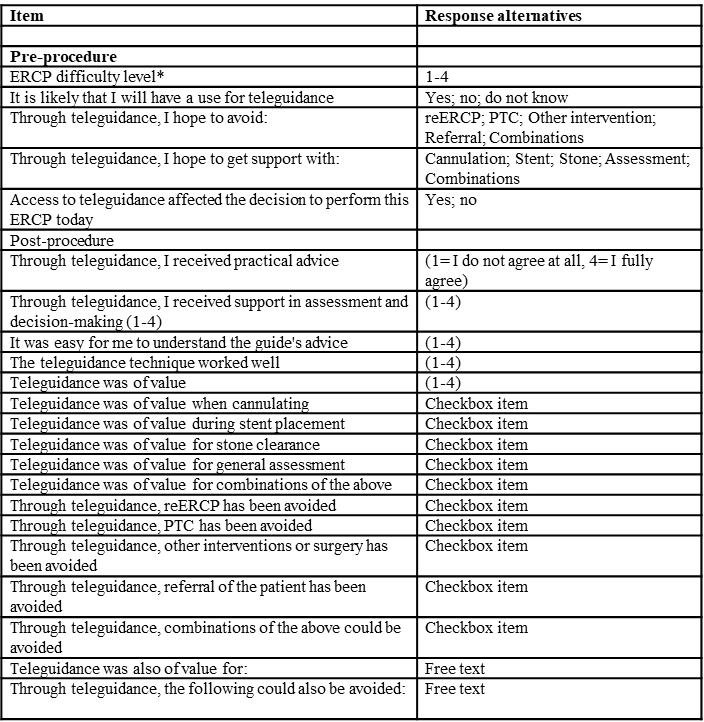

Supplement: Multimedia Appendix 2 [file humanfactors_v8i4e30867_app2.png]

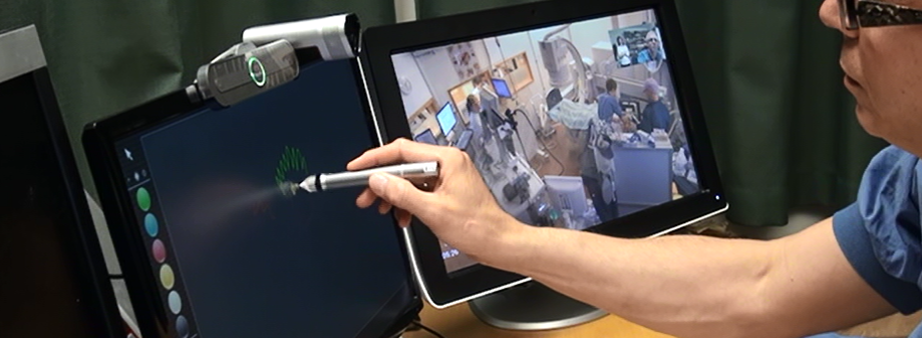

Supplement: Multimedia Appendix 3 [file humanfactors_v8i4e30867_app3.png]

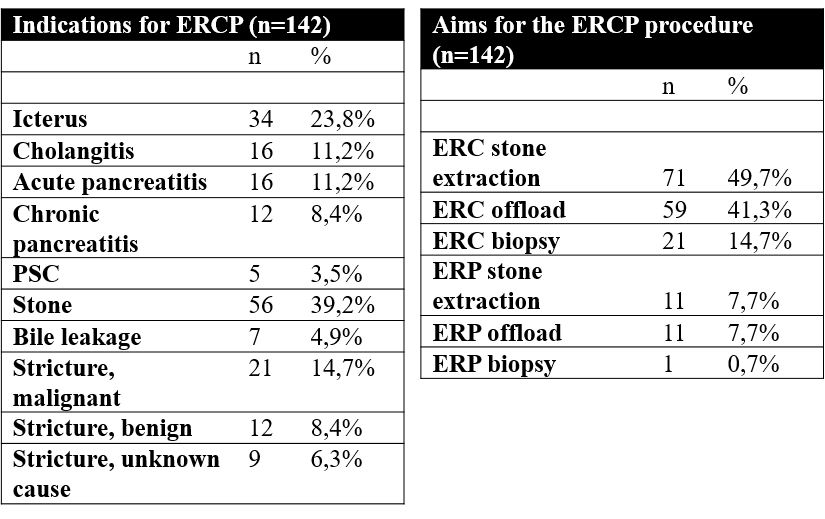

Supplement: Multimedia Appendix 5 [file humanfactors_v8i4e30867_app5.png]

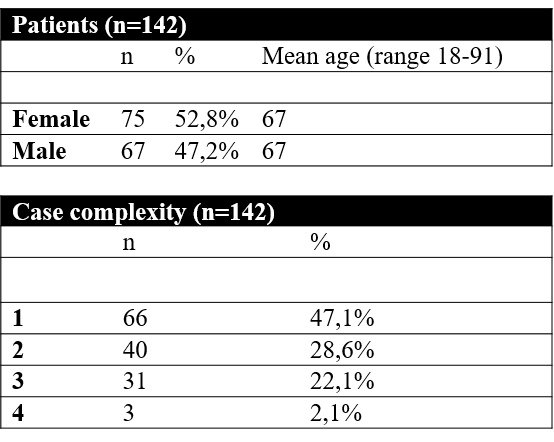

Supplement: Multimedia Appendix 6 [file humanfactors_v8i4e30867_app6.png]

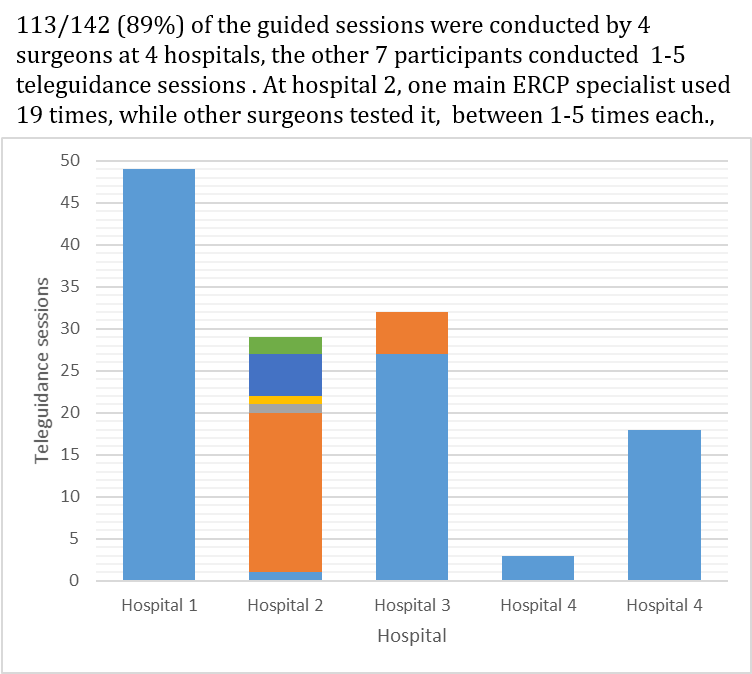

Supplement: Multimedia Appendix 7 [file humanfactors_v8i4e30867_app7.png]

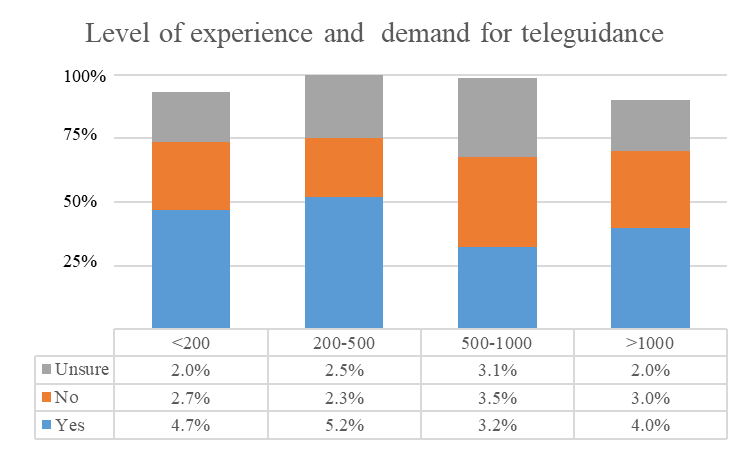

Supplement: Multimedia Appendix 8 [file humanfactors_v8i4e30867_app8.png]

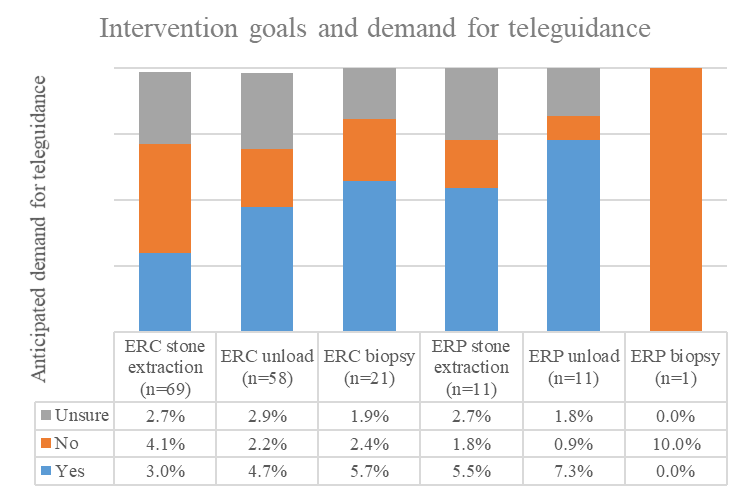

Supplement: Multimedia Appendix 9 [file humanfactors_v8i4e30867_app9.png]

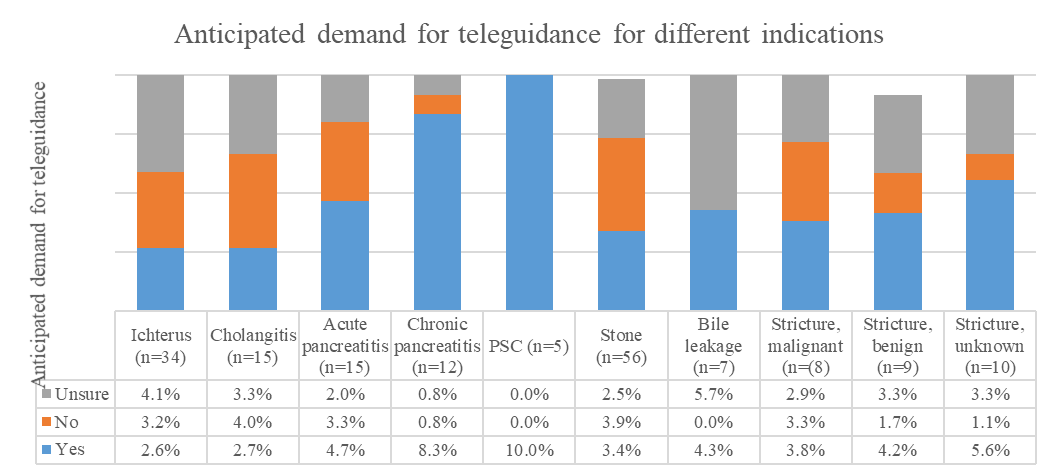

Supplement: Multimedia Appendix 10 [file humanfactors_v8i4e30867_app10.png]

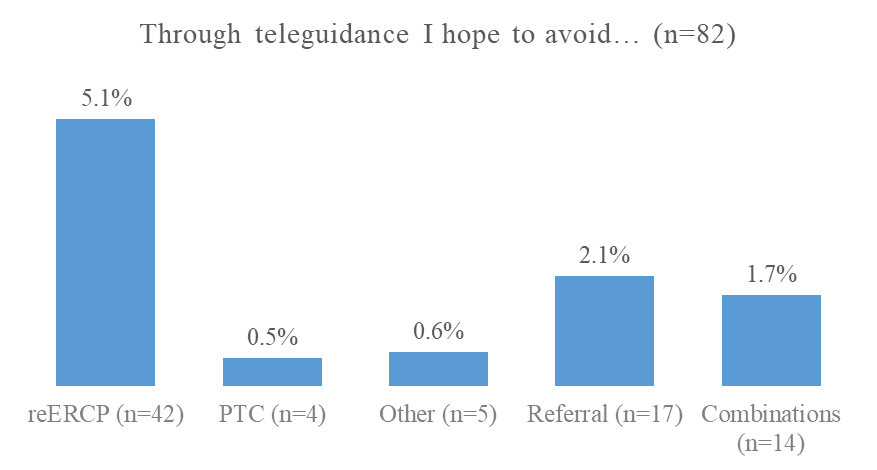

Supplement: Multimedia Appendix 11 [file humanfactors_v8i4e30867_app11.png]

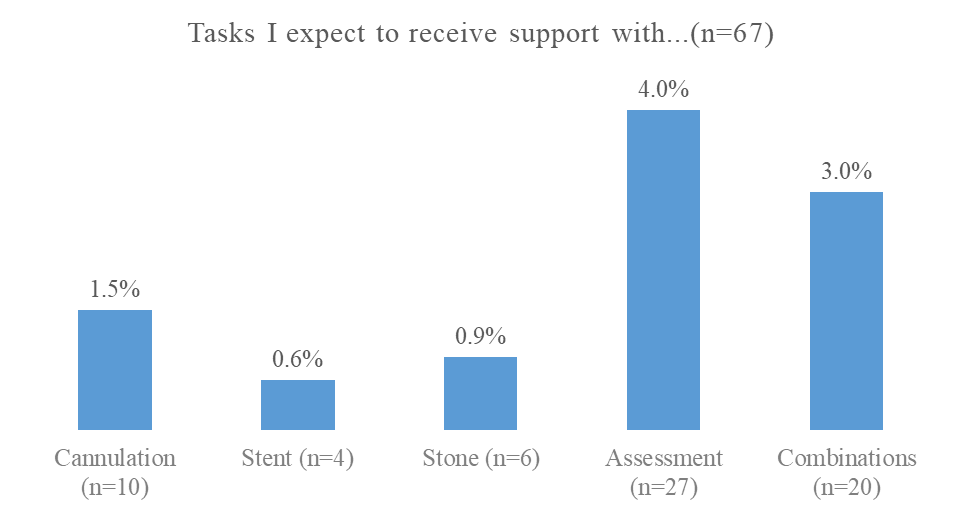

Supplement: Multimedia Appendix 12 [file humanfactors_v8i4e30867_app12.png]

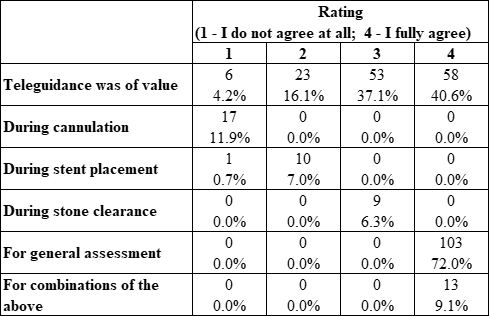

Supplement: Multimedia Appendix 13 [file humanfactors_v8i4e30867_app13.png]

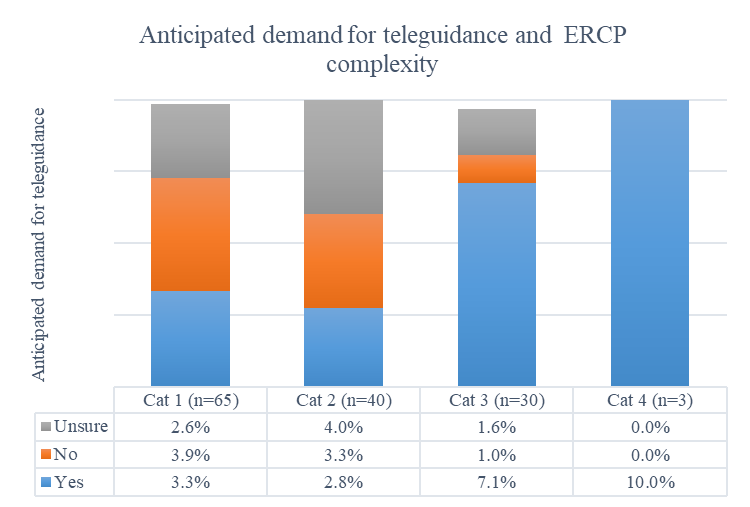

Supplement: Multimedia Appendix 14 [file humanfactors_v8i4e30867_app14.png]

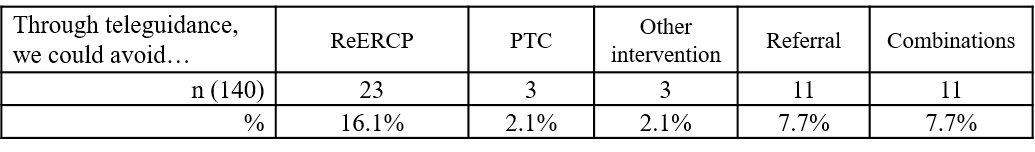

Supplement: Multimedia Appendix 15 [file humanfactors_v8i4e30867_app15.png]
