# Supplementary material for: User Experience in Remote Surgical Consultation: Survey Study of User Acceptance and Satisfaction in Real-Time Use of a Telemedicine Service
Source: JMIR Hum Factors. 2021 Nov 30;8(4):e30867. doi: 10.2196/30867 (PMC8672288; doi:10.2196/30867)
Supplement: Multimedia Appendix 4 [file humanfactors_v8i4e30867_app4.pdf]

Checklist for verification of function, teleguidance

Contents

1. Purpose of the checklist 1
2. Instruction 1
   2.1. Approval 1
   2.2. Checkpoints 1
   2.3. System startup 1
   2.4. Call the test room and possibly the remote site 1
   2.5. Image quality and image section 2
   2.6. Check lighting 2
   2.7. Sound level 2
3. Checklist: Verification before ERCP session 3

1. Purpose of the checklist

The purpose of the checklist is to enable the user at the clinics to be to carry out a quick routine with the most important steps to check that the system works prior to a teleguidance session. This checklist thus refers to the system and communication for the central clinic. If everything works smoothly when starting the system, simply sign the checklist.
The checkpoints are intended to support the user, further support can be found in these instructions. If something does not work and you do not manage to solve the problems yourself, call the support number at the bottom of the checklist.

2. Instruction

2.1. Approval

The person performing the check signs the checklist. Name, signature, date and whether the system was approved or not are stated. If there is a remark, state this under the remark or in a separate appendix. Enter attachment number.

2.2. Checkpoints

Here, all points are described in more detail for reference.

2.3. System startup

Central location: Does the system has electricity at all? Do the screens work? Is the network cable in? Are there warning messages on the video system? (Signifying, for example, a lack of network connections).

2.4. Call the test room and possibly the remote site

Central site: Call the central test system (Sjunet test room) to verify your own function. The number is 70101. It controls both communication from the hospital and into the county council's internal network (SJUNET).

Answer if the remote site calls. Does the network work at all, e.g. is it possible to get in touch through audio and video? Sound should work in both directions.
If a remote site calls, an endoscope image, X-ray image and room image should be shown. However, all three are not visible at the same time, only two images can be seen in parallel. The X-ray image will be displayed and it should be possible to switch between endoscope image and an overview image. The remote site will see a picture of the guiding surgeon.
At the central site, the light spectrum on the endoscope image should be checked, as well as the X-ray image (if in doubt, contact the surgeon who will provide the consultation) and the overview image that makes it possible to see how the patient is lying and how the surgeon and other team members are standing.

2.5. Image quality and image section

When a remote site is connected: One party describes what is shown on the endoscope image, including what is visible at the edges. The other counterparty checks agreement.
The remote site verifies that imagery from medical equipment on the local monitors corresponds with the transmitted images. For example, the shape of the endoscopic image should be round on all screens. If the images section does not match, an assessment is should be made as to whether the session still can be carried out. Deviations are to be documented.
Look at the video image. Do the movements chop, are they pixelated (small squares)? This may be due to a poor network connection. Check that the network connection is satisfactory by displaying the network speed. The deviation are to be documented

2.6. Check lighting

Is the room lighting set in a way that benefits or disturbs the imagery? Is it possible to change angles or light without disturbing the procedure and improve the video images?

2.7. Sound level

Are there any problems with the sound level? Too low or too high? Is it clear what the counterpart is saying? Take into account that different people may have different requirements for sound.

3. Checklist: Verification before ERCP session

Approval: The system's function and safety are checked and approved

Yes ☐

No, the system cannot be used, see note ☐

Control completed by	Sign.	Date

Checkpoints

1 System startup ☐
2 Dial-up the video conferencing parties ☐
3 Image quality and image section ☐
4 Lighting quality ☐
5 Sound level ☐

SUPPORT: (telephone number)

If error at startup, write down what is wrong. Please note measures to correct errors / settings. Refer to relevant point:
